# Effects of Insulin Lispro Mix 25 and Insulin Lispro Mix 50 on Postprandial Glucose Excursion in Patients with Type 2 Diabetes: A Prospective, Open-Label, Randomized Clinical Trial

**DOI:** 10.1007/s13300-018-0398-0

**Published:** 2018-03-08

**Authors:** Wei Li, Fan Ping, Lingling Xu, Meicen Zhou, Hongmei Li, Yaxiu Dong, Yuxiu Li

**Affiliations:** 1 Department of Endocrinology, Key Laboratory of Endocrinology of National Health and Family Planning Commission, Peking Union Medical College Hospital, Chinese Academy of Medical Sciences and Peking Union Medical College, Beijing, China; 2 Department of Endocrinology, China Meitan General Hospital, Beijing, China

**Keywords:** Postprandial glucose, Premixed insulin, Type 2 diabetes mellitus

## Abstract

**Introduction:**

We compared the effects of insulin lispro mix 25 (LM25) and insulin lispro mix 50 (LM50) on postprandial glucose excursion in patients with type 2 diabetes mellitus (T2DM).

**Methods:**

In this randomized, open-label, investigator-initiated trial, 81 T2DM patients treated with premixed human insulin 70/30 (PHI70/30) for more than 90 days were randomly divided into two groups and received a crossover protocol of either LM25 or LM50 twice daily for 16 weeks. Continuous glucose monitoring (CGM) was performed for 72 h at baseline and at the end of each treatment phase to evaluate glycemic excursions in the subjects.

**Results:**

The LM50 regimen resulted in significantly smaller postprandial glycemic excursions than the LM25 regimen after breakfast (1.3 ± 2.5 vs. 2.4 ± 2.6 mmol/L, *P* = 0.046) and dinner (1.5 ± 2.8 vs. 2.8 ± 2.4 mmol/L, *P* = 0.036). Glycosylated hemoglobin levels were similar for the patients on the three regimens. The percentage of patients who achieved their glycosylated hemoglobin target was significantly higher for the LM25 and LM50 regimens than for the PHI70/30 regimen, regardless of whether the target was set at 7.0% or 6.5%. The proportion of the patients who were hypoglycemic for a high percentage (> 10%) of the time was lower for the LM50 regimen than for the LM25 and PHI70/30 regimens.

**Conclusions:**

LM50 may provide better glycemic excursion control after breakfast and dinner than LM25 in T2DM patients.

**Trial Registration:**

http://www.chictr.org.cn # ChiCTR-TTRCC-12002516.

**Funding:**

Lilly Suzhou Pharmaceutical Co., Ltd. (Shanghai Branch, China) and National Key Program of Clinical Science of China (WBYZ 2011-873).

## Introduction

In recent years, the incidence of type 2 diabetes has been rapidly increasing in China; it had reached 11.6% in Chinese adults according to an epidemiological survey in 2010. As a result, China has become the nation with the largest number of diabetic patients [1]. The purpose of treating diabetes is to prevent the occurrence of complications. Epidemiological and intervention studies have demonstrated that glycosylated hemoglobin (HbA_1c_) is an independent risk factor for diabetic chronic complications. In addition, some studies have suggested that glucose excursions are closely related to the occurrence of complications [2–4].

When islet β-cell failure occurs in patients with type 2 diabetes, insulin therapy becomes inevitable. While insulin treatment can effectively reduce HbA_1c_ levels in patients, the pharmacokinetic characteristics of human insulin mean that it is sometimes difficult to avoid glycemic fluctuations and even hypoglycemia. Given the disadvantage of the slow onset of action of regular human insulin, a variety of fast-acting insulin analogues have been developed since the 1990s. Similarly, premixed insulin has developed from premixed human insulin to premixed insulin analogues. Several studies have demonstrated that postprandial glycemic control was improved and overall control was similar when T2DM patients were treated with a premixed insulin analogue rather than premixed human insulin [5–7]. Currently, there are two premixed insulin analogues available on the Chinese market, namely LM25 (Humalog^®^ Mix25, which contains 25% insulin lispro and 75% insulin lispro protamine) and LM50 (Humalog^®^ Mix50, which contains 50% insulin lispro and 50% insulin lispro protamine). However, there has been limited research into the effects of LM25 and LM50 on glucose control and glycemic excursions [8–10].

Compared to the self-monitoring of blood glucose (SMBG) using a fingerstick, continuous glucose monitoring (CGM) provides the opportunity to collect comprehensive information on both glycemic control and variability over the course of a day (24 h) and information on hypoglycemic episodes that the patient is unaware of (due to a lack of symptoms or because they are asleep at the time) [11]. Some studies have shown that glycemic excursion parameters evaluated by CGM were significantly related to plasma markers (glycosylated albumin and 1,5-anhydroglucitol) [12, 13].

Therefore, we conducted an open-label, randomized, crossover clinical trial to investigate the effects on glycemic control and excursions of switching from premixed human insulin 70/30 (PHI70/30) to LM25 or LM50 in T2DM patients with CGM. Among reported studies evaluating the effects of LM25 and LM50 on glycemic variability via CGM, our study included by far the largest number of subjects. The primary objective of this study was to evaluate the effects of LM25 and LM50 on postprandial glucose excursions, and the secondary objective was to compare the effects of the two premixed insulin analogues and PHI70/30 on postprandial glucose excursions, glycosylated hemoglobin, body weight, and hypoglycemia.

## Methods

### Study Design

**Fig. 1:**
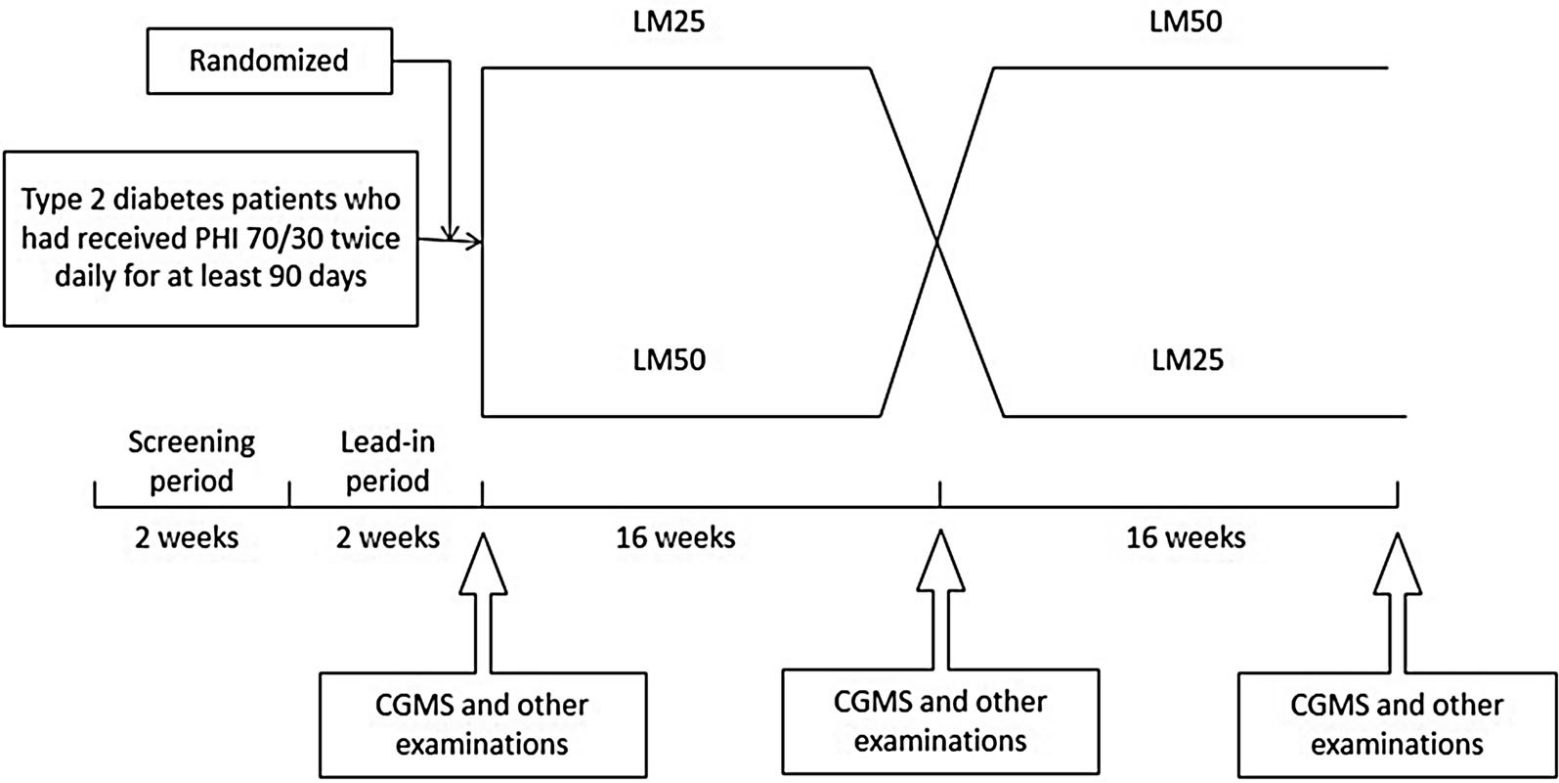
Subject flow diagram. *PHI70/30* premixed human insulin 70/30, *LM25* insulin lispro mix 25, *LM50* insulin lispro mix 50, *CGMS* continuous glucose monitoring system

This study was a randomized, open-label, crossover, controlled, investigator-initiated clinical trial, and is registered with http://www.chictr.org.cn (registration number: ChiCTR-TTRCC-12002516). The study comprised a 2-week screening period, a 2-week lead-in period, and two 16-week crossover treatment periods. The study design is shown in Fig. 1.

The subjects continued to receive the PHI70/30 regimen during a 2-week lead-in period, but were then randomly divided into two treatment arms: one arm received the LM25 regimen while the other arm received the LM50 regimen during the first 16-week treatment period, and the two arms then switched regimens for the subsequent 16-week treatment period.

All procedures were designed and performed in accordance with the ethical standards of the responsible committee on human experimentation (institutional and national) and with the Helsinki Declaration of 1964, as revised in 2013. Informed consent was obtained from all patients included in the study.

### Subjects

Only T2DM patients who were > 18 years old, had received PHI70/30 twice daily for at least 90 days (including in combination with oral hypoglycemic agents), had a recently measured HbA_1c_ ≥ 6%, and had visited the Department of Endocrinology at Peking Union Medical College Hospital from November 1st, 2013 to May 31st, 2015, were included in the study, provided that they did not meet any of the following exclusion criteria: patients with clinically significant hepatic dysfunction (alanine transaminase or alkaline phosphatase ≥ 2.5 times the upper limit of the normal reference value); patients with renal insufficiency (male serum creatinine ≥ 133 μmol/L, female serum creatinine ≥ 110 μmol/L); patients with diseases that may cause blood glucose elevation, such as an infection or hyperthyroidism; patients receiving glucocorticoid or estrogen treatment that could affect glucose metabolism; and patients with an inability to eat normally.

### Study Procedures and Treatment

The medical history (age, gender, duration of diabetes) was collected from each subject. The subjects continued to receive the PHI70/30 regimen during the 2-week lead-in period, and were then randomly divided into two treatment arms: one arm received the LM25 regimen and the other arm received the LM50 regimen during the first 16-week treatment period, and the two arms then switched regimens for the next 16-week treatment period. The first four weeks of each 16-week treatment phase were the insulin dose titration period, when the patients were asked to self-monitor their blood glucose levels and insulin dose adjustments were made based on their results (the adjustment protocol is shown in Table 1). The next 12 weeks were the steady dose period. Oral hypoglycemic agents were not adjusted throughout the entire period of the study. The targets for glycemic control were blood glucose levels before breakfast and dinner of > 3.9 and ≤ 6.1 mmol/L, respectively.

**Table 1:** Insulin sliding scale

Blood glucose (mmol/L)	Insulin dose adjustment
≤ 3.9	− 1 ~ 2 U
> 3.9 and ≤ 6.1	Unchanged
> 6.1 and ≤ 7.8	+ 1 ~ 2 U
> 7.8	+ 2 ~ 4U

During the study, the subjects were assigned diets recommended by nutritionists based on the 2013 China Medical Nutrition Therapy Guidelines for Diabetes [14]. The diets led to 45–60% of the total daily energy being derived from carbohydrates, 25–35% from dietary fat, and 15–25% from protein. The total daily energy intake was calculated based on the height and weight of each subject. Subjects were also required to exercise regularly during the study. During the 3 days of CGM at the end of each treatment phase, the subjects were given high-carbohydrate test meals (with 56.8–58.4% of the total energy deriving from carbohydrate, 15.7–17.2% from protein, and 24.4–26.7% from fat) on day 1 and high-fat test meals (with 39.8–40.7% of the total energy deriving from carbohydrate, 23.0–24.4% from protein, and 33.0–36.1% from fat) on day 2. They continued their habitual diets in day 3.

### Outcome Measures

Anthropometric parameters (height, weight, waist circumference, blood pressure) at baseline and at the end of each treatment phase were recorded, as well as the total daily insulin dose. In addition, various indicators (including HbA_1c_ and glycosylated albumin) were also measured, along with 72 h CGM. CGM was conducted using the CGM system GOLD (Medtronic). This system can measure the blood glucose level every 10 s, record the average value every 5 min, and record 288 measurements daily. The mean blood glucose levels 1 h before all three meals and 1 and 2 h after all three meals as well as the glycemic excursions after all three meals (postprandial glucose excursion, PPGE) were measured based on the meal time of each subject. Furthermore, the mean blood glucose during the night (0:00 a.m.–6:00 a.m., 22:00 p.m.–24:00 p.m.), the mean blood glucose during the day (6:00 a.m.–22:00 p.m.), the mean blood glucose throughout the whole day, the large amplitude of glycemic excursion (LAGE, difference between the highest and lowest blood glucose values during the 24 h monitoring period by CGMS), mean amplitude of glycemic excursion (MAGE, the mean value of the valid blood glucose excursion amplitudes that were greater than the standard deviation for all the glucose excursions during the given day), the percentage of time spent hypoglycemic during the whole day (percentage of time with blood glucose ≤ 3.9 mmol/L), the percentage of time spent hypoglycemic during the daytime (percentage of time with blood glucose ≤ 3.9 mmol/L between 6:00 a.m.–22:00 p.m.), the percentage of time spent hypoglycemic during the night (percentage of time with blood glucose ≤ 3.9 mmol/L between 0:00 a.m.–6:00 a.m. and 22:00 p.m.–24:00 p.m.), and the frequency of hypoglycemia (number of subjects with blood glucose ≤ 3.9 mmol/L excursions during a particular day, where blood glucose ≤ 3.9 mmol/L to > 3.9 mmol/L was counted as one hypoglycemic event) were also measured and recorded. To avoid the effects of nutritional intake changes on the blood glucose, only the CGM data for day 3 were collected for analysis in this paper. Other data will be published in other articles.

### Statistical Analysis

Measurement data were expressed as the mean ± SD, and count data were expressed as median (5–95% percentile). Measurement data were analyzed using the pairwise *t* test or an ANOVA, whereas count data were analyzed via the McNemar test. All statistical analysis was conducted using the SPSS 22.0 software.

## Results

### Subject Baseline Characteristics

**Fig. 2:**
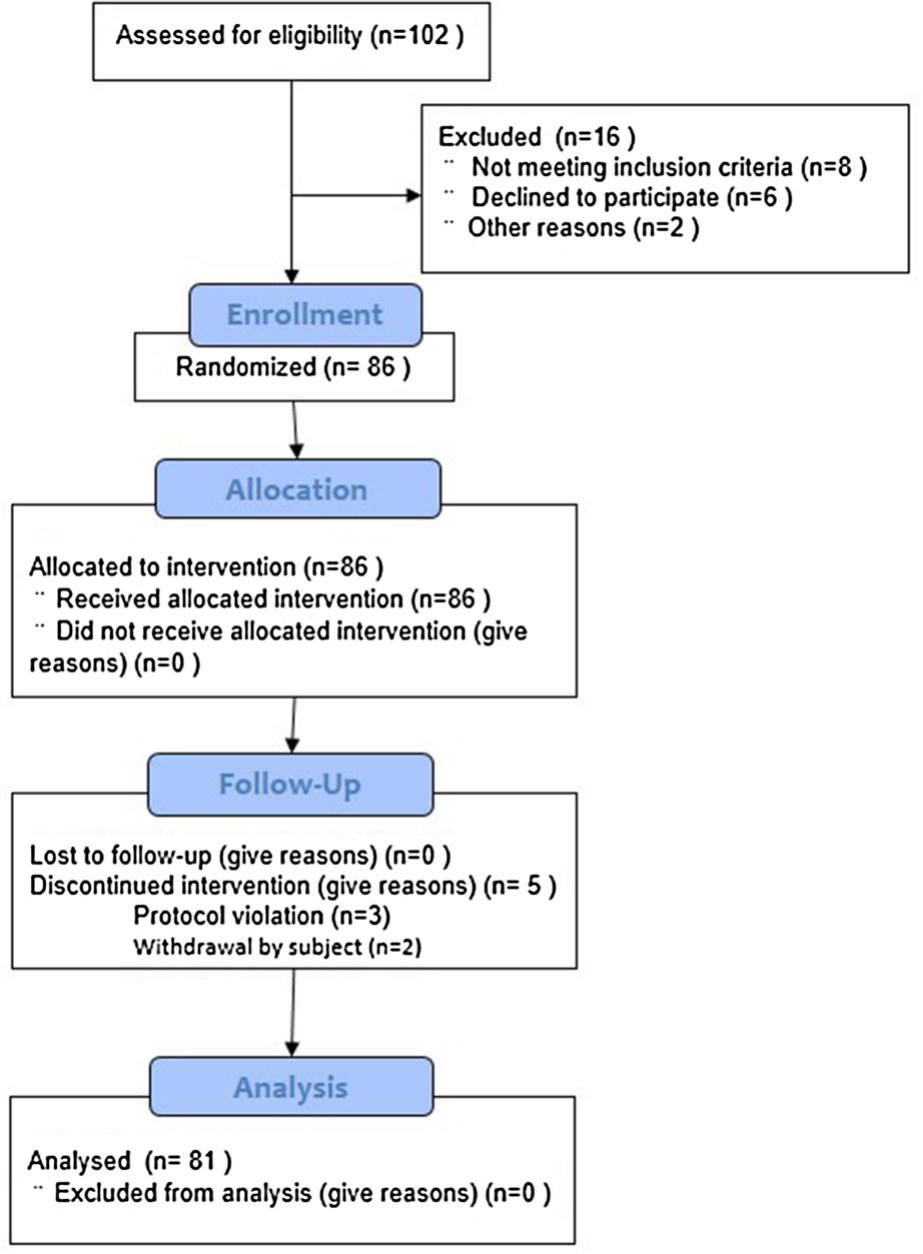
Patient disposition

Among the 86 subjects included in this study, 3 subjects were asked to withdraw from the study because they violated the study protocol (voluntary termination of oral hypoglycemic agents and insulin during the study period), 2 subjects voluntarily withdrew from the study, and 81 subjects completed the study (Fig. 2). The demographics and baseline characteristics of the subjects are shown in Table 2.

**Table 2:** Demographics and baseline characteristics of the subjects

Gender (male/female) (*n*)	31/50
Age (years)	59.9 ± 10.3
Duration of diabetes (years)	14.7 ± 8.4
Height (cm)	162 ± 20
Weight (kg)	68.7 ± 14.8
Waist circumference (cm)	87.0 ± 19.4
Systolic blood pressure (mmHg)	122 ± 13
Diastolic blood pressure (mmHg)	72 ± 9
Total daily insulin dose (IU)	40.2 ± 15.4
Subjects with combined oral hypoglycemic agents (%)	73 (90.1%)
Type of combined oral hypoglycemic agents (%)	
Metformin	50 (68.5%)
Acarbose	29 (39.7%)
Thiazolidinediones	6 (8.2%)

Data are given as mean ± SD

### Overall Glycemic Control

**Fig. 3a–b:**
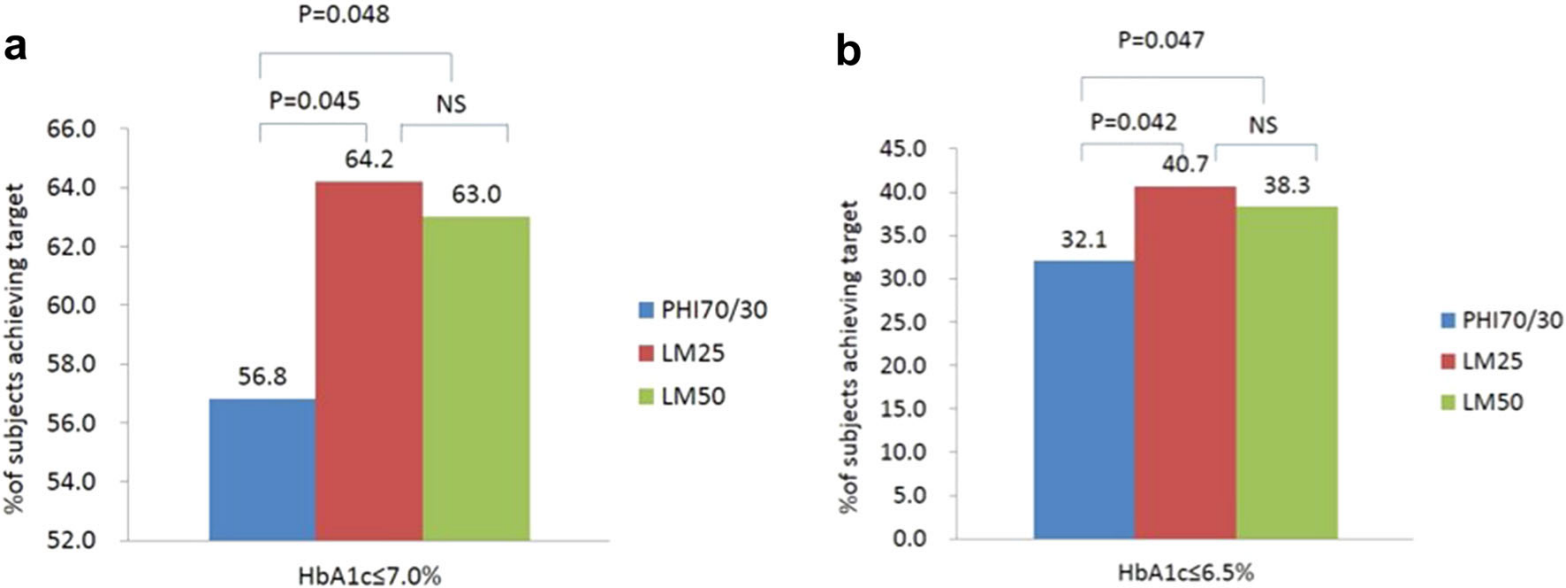
Percentage of the subjects who achieved a target HbA1c level of ≤ 7.0% (**a**) or ≤ 6.5% (**b**). *NS* not significant

No significant difference in glycosylated hemoglobin and glycosylated albumin levels was observed among three regimens (*P* > 0.05) (Table 3). On the other hand, the proportion of patients that achieved a glycated hemoglobin target of ≤ 7.0% was significantly higher for both the LM25 regimen (64.2%) and the LM50 regimen (63.0%) than for the PHI70/30 regimen (56.8%). Similarly, the proportion of patients that achieved a glycated hemoglobin target of ≤ 6.5% was significantly higher for both the LM25 regimen (40.7%) and the LM50 regimen (38.3%) than for the PHI70/30 regimen (32.1%) (Fig. 3).

**Table 3:** The effects of the three regimens on overall glycemic control

	PHI70/30	LM25	LM50	*P*_1_	*P*_2_	*P*_3_
HbA_1c_ (%)	7.8 ± 1.4	7.6 ± 1.1	7.5 ± 1.1	0.203	0.091	0.682
GA (%)	19.6 ± 3.9	19.6 ± 3.8	19.2 ± 3.9	0.936	0.515	0.572
Mean blood glucose (mmol/L) during the whole day	9.5 ± 2.5	9.6 ± 2.0	9.3 ± 2.6	0.768	0.687	0.500
Mean blood glucose (mmol/L) during the daytime (6:00 a.m.–22:00 p.m.)	10.6 ± 2.6	10.7 ± 2.5	10.2 ± 2.7	0.795	0.462	0.352
Mean blood glucose (mmol/L) during the night (0:00–6:00 a.m. and 22:00–24:00 p.m.)	8.4 ± 3.2	8.6 ± 2.7	8.3 ± 2.8	0.788	0.842	0.528

Data are given as mean ± SD or as median (5–95% percentile range)

*PHI70/30* premixed human insulin 70/30, *LM25* insulin lispro mix 25, *LM50* insulin lispro mix 50, *HbA*_*1c*_ glycated hemoglobin, *GA* glycated albumin, *PPGE* postprandial glucose excursion, *LAGE* large amplitude of glucose excursion, *MAGE* mean amplitude of glucose excursion

*P*_1_: PHI 70/30 vs. LM25, *P*_2_: PHI70/30 vs. LM50, *P*_3_: LM25 vs. LM50

There was no significant difference in mean blood glucose during the whole day, during the daytime (6:00 a.m.–22:00 p.m.), or during the night (0:00 a.m.–6:00 a.m., 22:00 p.m.–24:00 p.m.) among the three regimens (*P* > 0.05).

### Glycemic Excursions

Mean blood glucose at 2 h after breakfast was significantly lower in patients on the LM50 regimen than in those on the PHI70/30 regimen (9.5 ± 2.8 vs. 10.4 ± 3.2 mmol/L, *P* = 0.042). On the other hand, PPGE after breakfast was markedly lower in patients on the LM25 and LM50 regimens than in those on the PHI70/30 regimen (2.4 ± 2.6 vs. 3.8 ± 3.0 mmol/L, *P* = 0.005, and 1.3 ± 2.5 vs. 3.8 ± 3.0 mmol/L, *P* = 0.000, respectively), and lower in patients on the LM50 regimen than in those on the LM25 regimen (1.3 ± 2.5 vs. 2.4 ± 2.6 mmol/L, *P* = 0.046). There was no significant difference in mean blood glucose levels 1 h before breakfast and 1 h after breakfast among the three regimens (*P* > 0.05).

The mean blood glucose 1 h before lunch was significantly lower in patients on the LM50 regimen than in those on the PHI70/30 regimen (8.2 ± 3.3 vs. 9.5 ± 3.4 mmol/L, *P* = 0.022). There was no significant difference in mean blood glucose levels 1 and 2 h after lunch and in PPGE after lunch among the three regimens (*P* > 0.05).

**Fig. 4:**
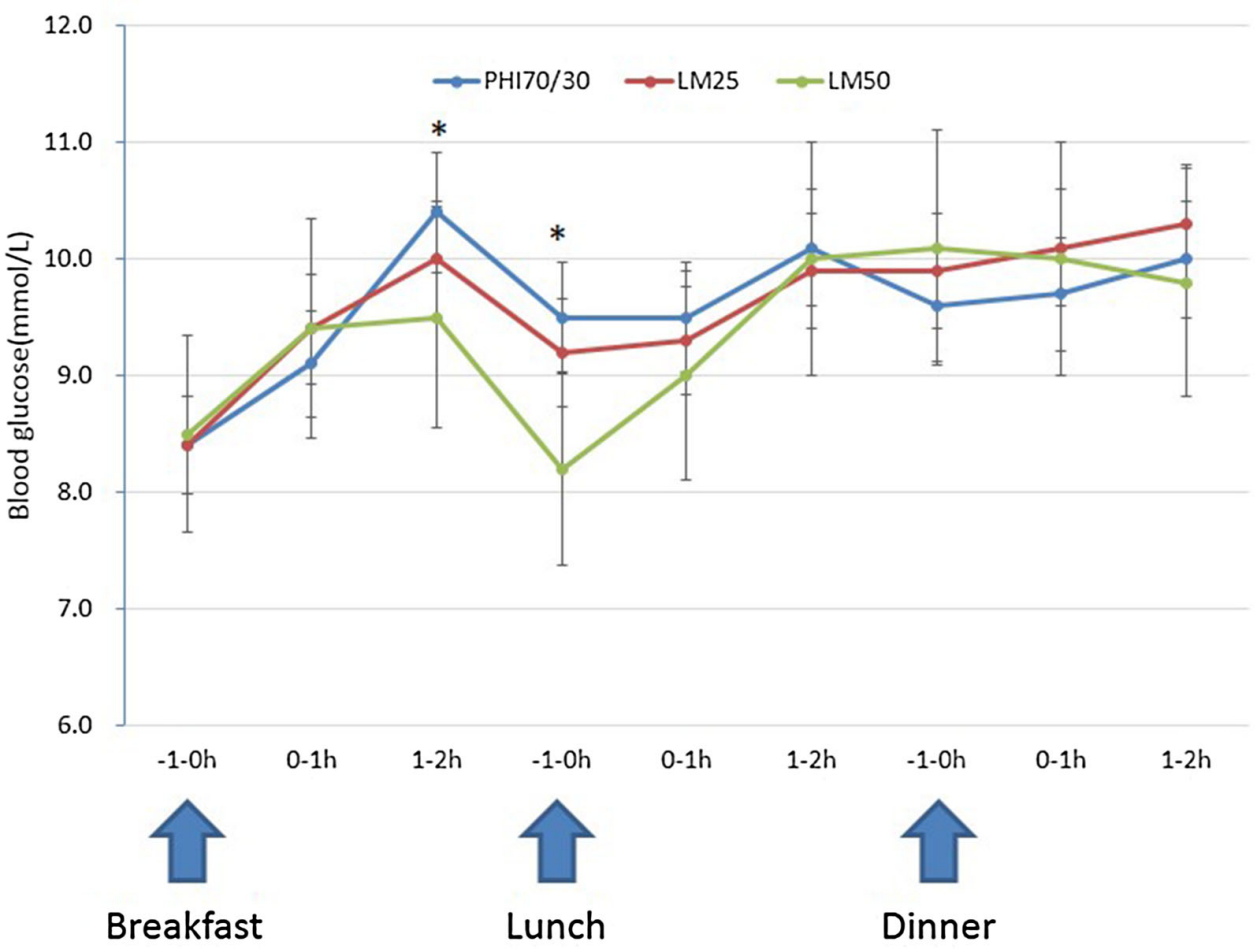
Blood glucose levels at different time points around three meals. *P < 0.05 (PHI70/30 vs. LM50)

**Fig. 5a–c:**
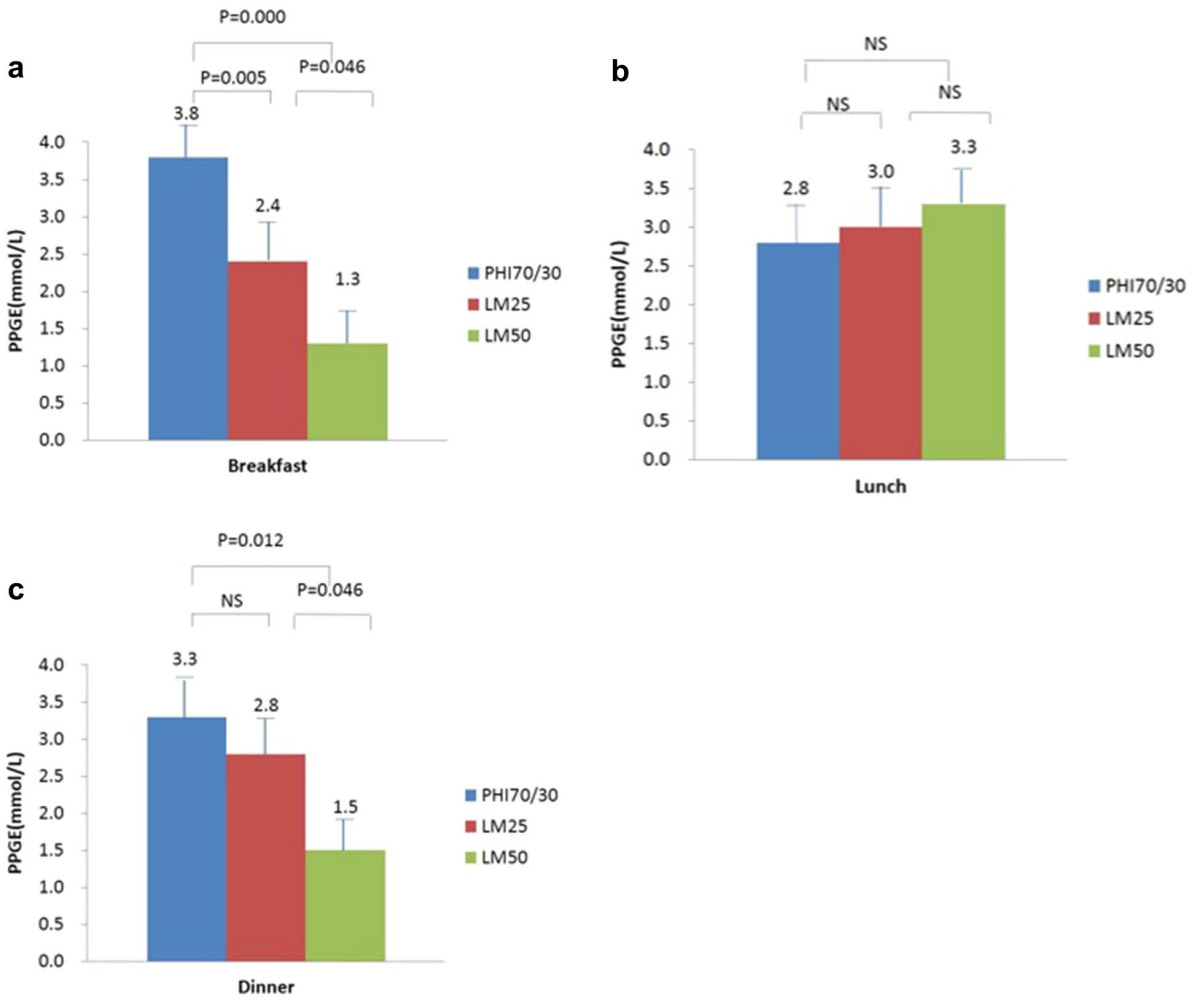
Postprandial glucose excursions following three meals: **a** breakfast, **b** lunch, **c** dinner. *NS* not significant, *PPGE* postprandial glucose excursion

While PPGE after dinner was significantly lower in patients on the LM50 regimen than in those on the PHI70/30 and LM25 regimens (1.5 ± 2.8 vs. 3.3 ± 3.4 mmol/L, *P* = 0.012, and 1.5 ± 2.8 vs. 2.8 ± 2.4 mmol/L, *P* = 0.036, respectively), there was no significant difference in PPGE after dinner between patients on the LM25 regimen and those on the PHI70/30 regimen. Further, there was no significant difference in mean blood glucose levels 1 h before dinner and 1 and 2 h after dinner among the three regimens (*P* > 0.05). Furthermore, there was no significant difference in LAGE and MAGE among the three regimens (*P* > 0.05) (Table 4, Figs. 4, 5).

**Table 4:** The effects of the three regimens on glycemic excursions

	PHI70/30	LM25	LM50	*P*_1_	*P*_2_	*P*_3_
Blood glucose at breakfast (mmol/L)						
1 h before meal	8.4 ± 2.9	8.4 ± 2.4	8.5 ± 2.5	0.971	0.849	0.828
1 h after meal	9.1 ± 2.8	9.4 ± 2.5	9.4 ± 2.5	0.587	0.572	0.979
2 h after meal	10.4 ± 3.2	10.0 ± 2.7	9.5 ± 2.8	0.445	0.042	0.384
PPGE	3.8 ± 3.0	2.4 ± 2.6	1.3 ± 2.5	0.005	0.000	0.046
Blood glucose at lunch (mmol/L)						
1 h before meal	9.5 ± 3.4	9.2 ± 3.0	8.2 ± 3.3	0.689	0.022	0.068
1 h after meal	9.5 ± 2.7	9.3 ± 2.8	9.0 ± 2.7	0.744	0.313	0.509
2 h after meal	10.1 ± 2.8	9.9 ± 2.9	10.0 ± 2.9	0.814	0.924	0.937
PPGE	2.8 ± 3.0	3.0 ± 2.9	3.3 ± 3.1	0.668	0.336	0.572
Blood glucose at dinner (mmol/L)						
1 h before meal	9.6 ± 3.5	9.9 ± 3.4	10.1 ± 3.2	0.592	0.378	0.737
1 h after meal	9.7 ± 3.0	10.1 ± 3.2	10.0 ± 2.8	0.428	0.552	0.848
2 h after meal	10.0 ± 3.2	10.3 ± 3.0	9.8 ± 2.8	0.574	0.686	0.383
PPGE	3.3 ± 3.4	2.8 ± 2.4	1.5 ± 2.8	0.325	0.012	0.036
LAGE (mmol/L)	9.3 ± 3.5	8.9 ± 3.1	8.9 ± 3.8	0.480	0.509	0.967
MAGE (mmol/L)	5.7 ± 2.7	5.6 ± 2.8	5.5 ± 2.8	0.872	0.715	0.849

Data are given as mean ± SD or as median (5–95% percentile range)

*PHI70/30* premixed human insulin 70/30, *LM25* insulin lispro mix 25, *LM50* insulin lispro mix 50, *HbA*_*1c*_ glycated hemoglobin, *GA* glycated albumin, *PPGE* postprandial glucose excursion, *LAGE* large amplitude of glucose excursion, *MAGE* mean amplitude of glucose excursion

*P*_1_: PHI 70/30 vs. LM25, *P*_2_: PHI70/30 vs. LM50, *P*_3_: LM25 vs. LM50

### Hypoglycemia

**Fig. 6:**
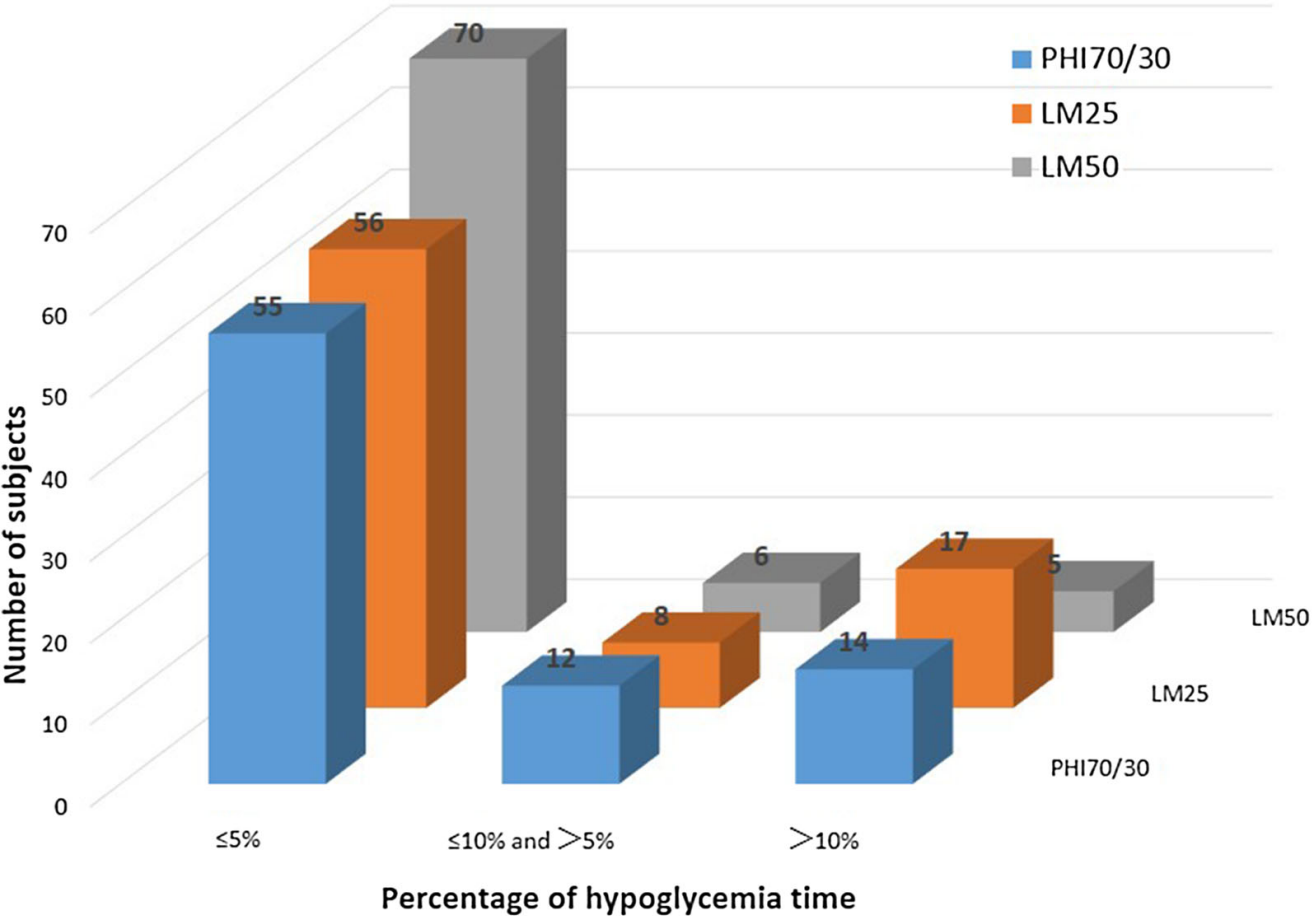
Frequency distributions of the percentage of time spent hypoglycemic during the whole day for patients on the three regimens. PHI70/30 vs LM25 vs LM50: *P* = 0.027; PHI 70/30 vs. LM25: *P*_1_ = 0.577; PHI 70/30 vs. LM50: *P*_2_ = 0.018; LM25 vs. LM50: *P*_3_ = 0.015. *PHI70/30* premixed human insulin 70/30, *LM25* insulin lispro mix 25, *LM50* insulin lispro mix 50

The subjects were divided into three groups according to the percentage of time that they spent hypoglycemic throughout the day (≤ 5%, ≤ 10% and > 5%, or > 10%). The frequency distribution of the percentage of time spent hypoglycemic during the whole day was then compared among the three insulin regimens (Fig. 6). The results showed that these frequency distributions differed significantly among the three insulin regimens (*P* = 0.027). There were also significant differences between the PHI70/30 and LM50 regimens (*P* = 0.018) and between the LM25 and LM50 regimens (*P* = 0.015) (adjusted significance level *α*′ = 0.017). In other words, a smaller proportion of the patients on the LM50 regimen spent a high percentage (> 10%) of their time hypoglycemic as compared to those on the LM25 and PHI70/30 regimens. Also, a higher proportion of the patients on the LM50 regimen spent low percentages (≤ 5%) of their time hypoglycemic as compared to those on the LM25 and PHI70/30 regimens.

### Insulin Dosage and Weight Gain

The total daily insulin doses as well as the insulin doses at breakfast and dinner were similar for the patients on the LM25 and LM50 regimens; mean daily doses of 42.3 and 40.8 IU, respectively, were reached at the end of each treatment phase. There was no significant difference in weight gained at the end of each treatment phase between the patients on the LM25 and LM50 regimens (Table 5).

**Table 5:** The effects of LM25 and LM50 on weight gain and insulin dosage

	LM25	LM50	*P*
Weight gain (kg)	1.6 ± 1.2	1.5 ± 1.2	0.867
Total daily insulin dose (IU)	42.3 ± 15.3	40.8 ± 15.4	0.651
Insulin dose at breakfast (IU)	21.3 ± 8.6	20.6 ± 8.5	0.628
Insulin dose at dinner (IU)	20.0 ± 7.6	20.2 ± 7.8	0.677

Data are given as the mean ± SD

*LM25* insulin lispro mix 25, *LM50* insulin lispro mix 50

## Discussion

The present study showed that the effects on the overall blood glucose control—such as HbA_1c_ and mean blood glucose during the whole day—were similar regardless of whether premixed human insulin or premixed insulin analogues were used and whether low-ratio premixed insulin analogues or high-ratio premixed insulin analogues were employed. This result is consistent with the results of the CLASSIFY study and our preliminary study [15, 16]. On the other hand, patients on both premixed insulin analogues were better at achieving the glycosylated hemoglobin target than those on premixed human insulin were. This may be a result of both reinforced blood glucose management and reduced glycemic excursions in the subjects. However, in contrast to what was seen in the CLASSIFY study, patients on LM50 and LM25 were found to be similarly good at achieving the glycosylated hemoglobin target. This inconsistency may be attributed to the different patient populations. Our study population was T2DM patients who had received PHI70/30 treatment (including when it was combined with oral hypoglycemic agents). Some of them achieved relatively good blood glucose control (baseline mean HbA_1c_ 7.8%), whereas the population of the CLASSIFY study was T2DM patients who had inadequate glycemic control with oral hypoglycemic agents (baseline mean HbA_1c_ 8.52–8.60%). It is often difficult to further improve the proportion of patients who achieve the target in populations with better blood glucose control.

As previously mentioned, the quality of blood glucose control—which is related to the occurrence of glycemic excursions and hypoglycemia—in patients with mild to moderate hyperglycemia should be improved in order to further reduce the risk of diabetes complications. Previous studies indicated that postprandial glycemic excursion was a major factor in metabolic imbalance in patients with mild to moderate hyperglycemia, and was associated with an increased risk of cardiovascular mortality [17, 18]. Therefore, postprandial glucose management was of great importance in these patients.

In this study, we obtained various parameters that described glycemic excursions in diabetic patients from different perspectives, such as PPGE, LAGE, and MAGE, by conducting CGM of the subjects. As a result, our study was able to obtain more comprehensive information regarding glycemic excursions in the patients than achieved in previous studies which used only 7-point self-monitoring of blood glucose (SMBG) as the evaluation indicator. Our results indicated that LM50 allowed better control over postprandial glycemic excursion than LM25, which may be related to the fact that a higher ratio of mealtime insulin was required to control the elevation of postprandial blood glucose in the Oriental population due to their carbohydrate-based diets. These findings are consistent with those obtained in the CLASSIFY study and other previous studies of the Japanese population [9, 10, 19]. Similarly, LM50 was better than PHI70/30 at controlling mean blood glucose 2 h after breakfast as well as the postprandial glycemic excursions following breakfast and dinner. Although LM25 has a relatively low proportion of prandial insulin, it was still better at controlling postprandial glycemic excursions following breakfast than PHI70/30 due to its fast action. Therefore, although they have similar effects on overall blood glucose, LM50 can facilitate a greater reduction in PPGE and improve the quality of blood glucose control in Chinese diabetic patients compared with LM25.

More T2DM patients in China are treated with premixed insulin than with basal plus prandial insulin [20]. At present, a considerable number of patients are still treated with premixed human insulin. Our study provided valuable information that should help those patients to optimize their insulin therapy. This was the difference between our study and the series of CLASSIFY studies that compared the effects of LM25 with those of LM50 as well as other studies that compared the effects of LM25 or LM50 with those of glargine plus lispro [15, 21–24].

Note that twice-daily premixed insulin analogue, whether LM25 or LM50, was not superior to PHI70/30 at decreasing PPGE after lunch. Performing conventional twice-daily injections of premixed human insulin before breakfast and dinner can reduce injection frequency, but this leads to poor post-lunch blood glucose control, which often requires patients to either control the amount of carbohydrate they consume during lunch or to use oral hypoglycemic agents too. A study by Nishimura et al. [19] comparing LM25 with LM50 found that neither twice-daily LM25 nor twice-daily LM50 provided sufficient post-lunch blood glucose control, indicating that thrice-daily premixed insulin analogues may be required to achieve better blood glucose control [25]. Therefore, post-lunch blood glucose monitoring should be reinforced in patients receiving twice-daily insulin analogue treatment, and prandial medication adjustments should be made for those that cannot achieve optimal blood glucose control.

Our study found that the percentage of the patients on the LM50 regimen who spent a high percentage (> 10%) of their time hypoglycemic was smaller than the corresponding percentages for the LM25 and PHI70/30 regimens. This may be attributed to the lower proportion of insulin lispro protamine in LM50. The total daily insulin doses and weight changes were similar for the patients on the LM25 and LM50 regimens. These results are consistent with those from the series of CLASSIFY studies.

We acknowledge that the relatively small number of patients included in this study is a possible limitation of it. The levels of glycosylated hemoglobin in this study population were relatively low, which makes it difficult to extend the conclusions drawn from these results to all T2DM patients. Also, the crossover design we used may have obscured the effects of the two insulins on patient weight. CGM data were obtained during only one day in each phase, so they represent limited information. However, the results of this study should still help to optimize insulin therapy.

## Conclusion

Our study showed that LM50 provides better control over post-breakfast and post-dinner blood glucose and postprandial glycemic excursions in T2DM patients with mild to moderate hyperglycemia who are receiving premixed human insulin, and thus improves the quality of blood glucose control.
